# Factors influencing health care and service providers’ and their respective “at risk” populations’ adoption of the Air Quality Health Index (AQHI): a qualitative study

**DOI:** 10.1186/s12913-016-1355-0

**Published:** 2016-03-31

**Authors:** Sally Radisic, K. Bruce Newbold

**Affiliations:** School of Geography and Earth Sciences, McMaster University, Hamilton, ON Canada; City of Hamilton Public Health Services, Hamilton, ON Canada

**Keywords:** Population health, Air pollution, Air Quality Health Index (AQHI), Diffusion of innovations theory, Socioeconomic status (SES)

## Abstract

**Background:**

The Air Quality Health Index (AQHI) provides air quality and health information such that the public can implement health protective behaviours (reducing and/or rescheduling outdoor activity) and decrease exposure to outdoor air pollution. The AQHI’s health messages account for increased risk associated with “at risk” populations (i.e. young children, elderly and those with pre-existing respiratory and/or cardiovascular conditions) who rely on health care and service providers for guidance. Using Rogers’ Diffusion of Innovations theory, our objective with respect to health care and service providers and their respective “at risk” populations was to explore: 1) level of AQHI knowledge; 2) factors influencing AQHI adoption and; 3) strategies that may increase uptake of AQHI, according to city divisions and socioeconomic status (SES).

**Methods:**

Semi-structured face-to-face interviews with health care (Registered Nurses and Certified Respiratory Educators) and service providers (Registered Early Childhood Educators) and focus groups with their respective “at risk” populations explored barriers and facilitators to AQHI adoption. Participants were selected using purposive sampling. Each transcript was analyzed using an Interpretive Description approach to identify themes. Analyses were informed by Rogers’ Diffusion of Innovations theory.

**Results:**

Fifty participants (6 health care and service providers, 16 parents, 13 elderly, 15 people with existing respiratory conditions) contributed to this study. AQHI knowledge, AQHI characteristics and perceptions of air quality and health influenced AQHI adoption. AQHI knowledge centred on numerical reliance and health protective intent but varied with SES. More emphasis on AQHI relevance with respect to health benefits was required to stress relative advantage over other indices and reduce index confusion. AQHI reporting at a neighbourhood scale was recognized as addressing geographic variability and uncertainty in perceived versus measured air quality impacting health. Participants predominantly expressed that they relied on sensory cues (i.e. feel, sight, taste) to determine when to implement health protective behaviours. Time constraints were identified as barriers; whereas local media reporting and wearable devices were identified as facilitators to AQHI adoption.

**Conclusion:**

Increasing knowledge, emphasizing relevance, and reporting AQHI information at a neighbourhood scale via local media sources and wearable devices may facilitate AQHI adoption while accounting for SES differences.

## Background

Air pollution is detrimental to public health and particularly to the “at risk” population including young children [1], seniors (≥65 years) [2] and individuals with existing respiratory and/or cardiovascular conditions [3] since it can adversely impact respiratory and cardiovascular systems [4–6]. The World Health Organization (WHO) estimated that 3.7 million people around the world died in 2012 as a result of outdoor air pollution exposure [7]. In Canada, between 2008 and 2031, air pollution attributed deaths have been predicted to rise 83% [8]. In the Organization for Economic Co-operation and Development (OECD) countries, the economic costs of air pollution were estimated to have reached 1.7 trillion dollars (US) in 2010 [9].

Therefore, strategies to protect the public from exposure to air pollution and adverse health effect are critical. The Air Quality Health Index (AQHI) is a risk communication tool developed to provide hourly air quality and health information such that the public can implement health protective behaviours, such as reducing and/or rescheduling outdoor activity and decrease exposure to outdoor air pollution [10]. The AQHI is a relatively easy to understand 10-point scale (low risk 1-3, medium risk 4-6, high risk 7-10, very high risk greater than 10) [10] which incorporates health messages according to health risk categories and accounts for the increased risk of “at risk” populations as presented in Table 1 [10].

**Table 1:** Air Quality Health (AQHI) messages according to health risk categories [10]

Health Risk	Air Quality Health Index	Health Messages	
		At Risk Population^a^	General Population
Low	1 - 3	Enjoy your usual outdoor activities.	Ideal air quality for outdoor activities.
Moderate	4 - 6	Consider reducing or rescheduling strenuous activities outdoors if you are experiencing symptoms.	No need to modify your usual outdoor activities unless you experience symptoms such as coughing and throat irritation.
High	7 - 10	Reduce or reschedule strenuous activities outdoors. Children and the elderly should also take it easy.	Consider reducing or rescheduling strenuous activities outdoors if you experience symptoms such as coughing and throat irritation.
Very High	Above 10	Avoid strenuous activities outdoors. Children and the elderly should also avoid outdoor physical exertion.	Reduce or reschedule strenuous activities outdoors, especially if you experience symptoms such as coughing and throat irritation

^a^People with heart or breathing problems are at greater risk

As a health promotion tool, AQHI reporting in the City of Hamilton started in summer 2011, although it had been introduced in the City of Toronto slightly earlier in 2008 [11]. In the City of Hamilton, promotion of the AQHI included the use of various media sources such as television, newspaper, radio, transit shelters, billboards and website. As an employee of the City of Hamilton’s Public Health Services, the first author (SR) participated in face-to-face outreach to promote the AQHI to the public including the at risk population by attending local festivals and fairs held throughout the city. Moreover, AQHI promotional material was delivered either in person by the first author and/or via mail to both health care and service providers with the responsibility of caring for at risk populations and included: child care facilities, retirement homes, respiratory health clinics, recognizing that health care and service providers are regarded as the top source of health information [12]. Therefore, adoption of the AQHI by both health care and service providers and the at risk populations in their care is essential to the health protection of those at increased risk from exposure to air pollution. It is important to explore how AQHI information is used by health care and service providers and relayed to others, including at risk populations, and how receptive these different groups are to the new tool. In spite of this, the factors facilitating its uptake within Hamilton, or elsewhere, have not been explored to date, limiting understanding of how best to implement the tool.

Health behaviour theory places risk perceptions at its core [13]; therefore, with respect to AQHI adoption, perceptions of air quality and health are at the heart of this health protective behaviour. Moreover, diffusion of Innovations (DOI) theory [14] can be used to understand AQHI adoption by both health care and service providers and their respective at risk populations. In public health, diffusion of innovations has been used to better understand dissemination and implementation of interventions in various areas such as skin cancer [15], cardiovascular disease (CVD) [16], HIV/AIDS [17] and substance abuse [18]. However, concerns have been raised about the potential of diffusion of innovations to widen socioeconomic (SES) gaps which in turn increase health disparities in the population [14]. The theory maintains that adopters (i.e. health care and service providers and their respective at risk populations) decide whether to adopt an innovation (i.e. AQHI) by weighing the benefits and barriers of the new innovation (i.e. AQHI) [14].

**Fig. 1:**
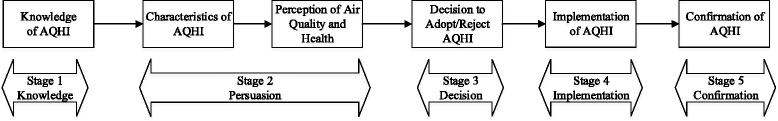
AQHI Adoption Process (Adapted from Rogers, [14])

Accordingly, DOI theory outlines a five stage process [14] (Fig. 1) that can be applied to AQHI adoption. The first stage is the knowledge stage which initiates the process; while the second stage is the persuasion stage which involves formation of a negative or positive attitude about the innovation (i.e. AQHI) via the perceived characteristics of the innovation including: relative advantage (degree to which the AQHI is better than the previous one), compatibility (degree to which the AQHI fits with existing values, past experiences and needs), complexity (degree to which the AQHI is perceived as being too difficult to understand and use), trialability (degree to which the AQHI can be experimented with before committing to using it) and observability (degree to which the results of using the AQHI are visible to adopters) [14].

The third stage is the decision stage where adoption or rejection of the innovation (i.e. AQHI) is considered, and the fourth stage is the implementation stage where the innovation (i.e. AQHI) is put into practice. The fifth stage is the confirmation stage, where reinforcement for the innovation-decision (i.e. adoption) already formed occurs.

Using Hamilton, Ontario as an example, and Rogers’ Diffusion of Innovations (DOI) theory to inform AQHI adoption, this paper explores: 1) level of AQHI knowledge; 2) factors influencing AQHI adoption and; 3) strategies that may increase uptake of AQHI with respect to health care and service providers and their respective “at risk” populations according to city divisions and SES.

## Methods

We used qualitative methods to bring forth more in-depth and contextualized meanings that are connected to the risk and the role of everyday experience in how people understand air pollution which the typical quantitative questionnaire-based approach fails to capture [19].

An Interpretive Description qualitative approach as described by Thorne [20] guided research design and analysis. This inductive analytic approach emphasizes use by health professionals who are interested in developing applied health knowledge and bridging the research-practice gap.

### Ethical permissions and data trustworthiness

This research received ethics approval from McMaster University Research Ethics Board.

Additionally, an audit trail was used to document the steps taken throughout the duration of the study. All sessions were conducted by the first author (SR) who provided an overview of the study and the interview guide and reviewed ethical and procedural aspects for voluntary participation, audio recording, transcription and data validation. Participants were given the opportunity to ask questions about the research and each person completed a consent form prior to participating in the study. To increase trustworthiness of the results and establish credibility, transferability, dependability and confirmability we used: purposive sampling, member checking, triangulation, audio recorded data and an audit trail [21, 22].

### Setting

Located at the western end of Lake Ontario, the City of Hamilton, Ontario is an industrial city consisting of a population of over 519, 000 people in 2016, with 84.1% speaking English in the home [23]. Several studies have identified that there are spatial variations in air pollution concentrations in the City [24–26] with a number of factors contributing to this spatial variability including [26] vehicles/traffic, industry/facilities, meteorological conditions/atmospheric inversions, and the geography of the city which is divided into an ‘upper’ and ‘lower’ city divided by the Niagara Escarpment. This upper and lower city divide potentially entraps pollutants in the lower SES areas, below the Niagara Escarpment, and closer to the industrial core (IC). From this point on in the paper, lower SES refers to the area below the Niagara Escarpment and closer to the IC; while higher SES refers to the area above the Niagara Escarpment and further from the IC.

The City has experienced a demographic shift with wealthier individuals moving out of the lower city and into the suburban areas above the escarpment and to the west of the downtown core, leaving lower SES individuals in the inner lower City [27]. This pattern based on city divisions and SES has also been found in perceptions of air quality and health and incidence of adverse health conditions including respiratory related and cardiovascular related emergency room visits and certain cancers such as lung cancer [27–34].

### Study sample selection

Purposive sampling was used to select health care and service providers and at risk populations in both lower and higher SES neighbourhoods. The selection of health care and service providers and their respective at risk populations across lower and higher SES areas was designed to account for spatial variations in air pollution concentrations, differences in perception of air pollution and health and health disparities that exist according to city divisions and SES.

Potential interview participants including: Registered Nurses (RN) working in supervisory positions in retirement homes, RNs working as Certified Respiratory Educators (CRE) in respiratory health clinics and Registered Early Childcare Educators (ECE) working in supervisory positions in childcare facilities were contacted by phone. Those who showed an interest were either emailed an information sheet and consent form or they were hand delivered to respective work sites. Face-to-face interviews were scheduled based on the participants’ availability and conducted at each participant’s work site.

Focus group participants consisted of people with existing respiratory conditions, seniors (≥65 years) and parents of young children. Participants were recruited with the assistance of their respective health care and service providers at centres in both lower and higher SES areas. Participants either contacted the first author or their respective health care and service provider to confirm participation.

### Data collection

**Fig. 2:**
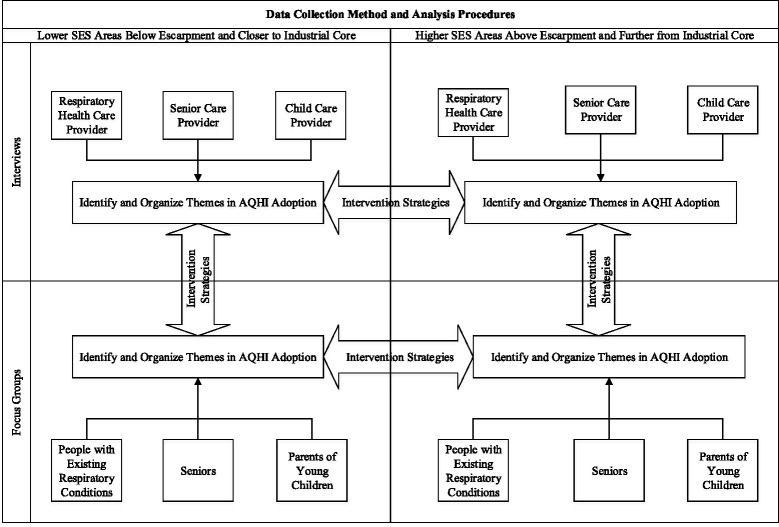
Data collection methods and analysis procedures

In order to compare AQHI adoption in the at risk populations with their respective health care and service providers’ adoption of the AQHI, data collection was conducted in two phases. The first phase consisted of interviews with health care and service providers while the second phase consisted of focus groups representing at risk populations (i.e. people with existing respiratory conditions, seniors and parents of young children); both phases included participants in lower and higher SES areas as presented in Fig. 2. The collection of data in this manner allowed for information to be generated by both groups such that any similarities and differences in AQHI knowledge, factors influencing AQHI adoption along with strategies to increase AQHI uptake could be explored.

Six interviews were conducted in October 2012. Interview participants consisted of supervisory staff including RNs working in licensed retirement homes, CREs working in respiratory health clinics and ECEs working in licensed childcare centres in both lower and higher SES areas. All interviews were conducted face-to-face at each of the respective worksites. Most lasted 30 minutes. The 6 interview participant characteristics are presented in Table 2.

**Table 2:** Interview participant characteristics

	*N* = 6 (%)
Gender	
Male	1 (17.0%)
Female	5 (83.0%)
Employee Status	
ECE, Supervisor Child Care Facility	2 (33.3%)
RN, Supervisor Senior Retirement Home	2 (33.3%)
RN, Certified Respiratory Educator	2 (33.3%)
At Risk Population Served	
Young Children	2 (33.3%)
Senior (≥65 years)	2 (33.3%)
Existing Respiratory Condition	2 (33.3%)
SES Area Served/Location	
Higher/Above Niagara Escarpment	3 (50.0%)
Lower/Below Niagara Escarpment	3 (50.0%)

Six focus groups were conducted between November 2012 and April 2015 ranging from 5 to 10 participants. The focus groups included representative members from each of the at risk populations from both lower and higher SES areas. Therefore, focus group participants consisted of people with existing respiratory conditions, seniors and parents of young children. All focus groups were conducted face-to-face in respiratory health clinics, public buildings, and recreation centres in Hamilton, and lasted about 1 hour. The 44 focus group participant characteristics are presented in Table 3.

**Table 3:** Focus Group Participant Characteristics

	*N* = 44 (%)
Gender	
Male	10 (23%)
Female	34 (77%)
Age	
18–24	5 (11%)
25–34	8 (18%)
35–44	3 (7%)
45–54	2 (4%)
55–64	6 (14%)
65–74	13 (30%)
75 and over	7 (16%)
Education	
Elementary School	1 (2%)
High School	19 (43%)
College	16 (36%)
University	8 (18%)
At Risk Group Represented	
Young Children	16 (36%)
Older Adults (≥65 years)	13 (30%)
Existing Respiratory Condition	15 (34%)
SES Area of Residence/Location	
Higher/Above Niagara Escarpment	21 (48%)
Lower/Below Niagara Escarpment	23 (52%)

### Interview/Focus group questions

The same questions were asked of the health care and service providers as well as the at risk populations, but the context was appropriately set with a parenthesis that included: “As a health care/service provider caring for people with exiting respiratory conditions/seniors/children or parent of a young child/senior/person with existing respiratory conditions…can you tell me from your perspective…” and then followed by the questions. Therefore, questions pertaining to AQHI knowledge included: *“Have you heard of the Air Quality Health Index (AQHI)?*” and *“Do you know where to check for daily Air Quality Health Index (AQHI)?”* Additionally questions exploring characteristics of the AQHI and potential barriers to adoption included: “*Do you check the Air Quality Health Index (AQHI)? Why or why not?”* and *“Do you follow the AQHI Health Messages which tell you when to consider reducing or re-scheduling outdoor physical activity? Why or why not?”* Furthermore, questions exploring perceptions of air quality and health included: *“Do you think the air in your neighbourhood affects your health? Why or why not?”* In order to explore facilitators to AQHI adoption and strategies to increase AQHI uptake, participants were asked: *“What do you think can be done to encourage/promote the use of the AQHI?”*

### Data analysis

According to Interpretative Description, data analysis involves four sequential cognitive processes: (1) comprehending everything one can about the setting and experiences of participants, (2) synthesizing instances or events to describe composite patterns, (3) theorizing to develop explanations for synthesized data, and (4) recontextualizing findings to other settings and contexts [20]. Each participant who agreed to be contacted was provided with a transcript of their session and was asked to validate the accuracy, clarity and completeness of the data and to mark passages they did not want quoted directly. NVivo10 (QSR International), a qualitative analysis software was used to organize, manage and code the validated interview and focus group data. We used constant comparison of interview data with other interview data and focus group data, theory and literature. New codes developed and evolved through the analysis.

## Results

Three broad categories evolved from analysis of the transcripts, including AQHI knowledge, factors influencing AQHI adoption and strategies to increase AQHI uptake. These categories, along with the various themes in each category, are summarized in Table 4 and further described with supportive quotes below.

**Table 4:** Themes Corresponding to AQHI Knowledge, Factors Influencing AQHI Adoption and Strategies Increasing AQHI Uptake

Category	Theme
AQHI Knowledge	Numerical Reliance
	Health Protective Intent
	SES Differences
Factors Influencing AQHI Adoption	Relevance
	Index Confusion
	Sensory Cue Precedence
	Time Constraints
Strategies Increasing AQHI Uptake	Professional Network Promotion
	Health Benefit Emphasis
	Neighbourhood Scale Focus
	Local Media Reporting
	Wearable Device Option

### AQHI knowledge

#### Numerical reliance

Participants expressed that AQHI knowledge centred on numerical reliance. When health and service care providers and their respective at risk populations described the AQHI, descriptions involved the use of numbers to either reflect risk or access to AQHI information. To highlight health risks due to air pollution exposure and differences within the population, the respiratory health care provider in the lower SES area indicated that*“…it may not bother somebody when it's[AQHI] at 6.”* Moreover, people with existing respiratory conditions in the higher SES area noted that AQHI numbers reflect risk and indicated that: *“The weather network website you can click right on it for risk for number air quality.”* Numerical reliance was also apparent in reference to accessing AQHI information. The child care provider in the lower SES area recalled that the AQHI could be accessed: *“… on the Channel 47”* and people with existing respiratory conditions in the higher SES area concurred that: *“The weather channel has it every 10 minutes.”*

#### Health protective intent

Participants also described the health protective intent of the tool. Health care and service providers described the AQHI as a health protection tool and identified that the AQHI could be used to protect the health of their respective at risk populations. The respiratory health care provider in the lower SES area indicated: *“Give them the tools for them to best manage their disease, go to the tools to avoid the triggers, smog is a trigger and we talk about it…”* Moreover, health protective intent of the AQHI was expressed by the child care provider in the higher SES area as follows: *“…check air quality to determine if any of our children that have asthma should be excluded from outdoor play and that kind of thing…”*

### SES differences

Through interview and focus group discussions, differences in AQHI knowledge according to SES were brought to light. Although respiratory health care providers in both lower and higher SES areas voiced AQHI knowledge, AQHI knowledge within their respective at risk populations varied with SES. People with existing respiratory conditions attending clinics in the higher SES area explained that AQHI information could be obtained on *“The Weather Channel.”* However, people with existing respiratory conditions attending clinics in the lower SES area indicated that *“People don’t even know what it [AQHI] is.”* Moreover, the senior care provider in the higher SES area explained that information about air quality was obtained from *“…the news and the weather…”* However, the senior care provider in the lower SES area indicated that with respect to the AQHI: *“This is an entirely new thing for me.”* This same pattern of AQHI knowledge was expressed by child care providers in higher and lower SES areas. The child care provider in the higher SES area indicated that AQHI knowledge was obtained from: *“I believe it was from the supervisor's network.”* On the other hand, the child care provider in the lower SES expressed novelty of the AQHI with the following comment: *“Oh so you do have a website for that?”* However, seniors in both higher and lower SES areas expressed lack of AQHI knowledge. Seniors in the higher SES area enquired: *“Is this tied in with your heat alerts?”* And seniors in the lower SES area indicated that they “…*have never seen that index”*.

### Factors influencing AQHI adoption

#### Relevance

Both health care and service providers and their respective at risk populations emphasized that the AQHI was not relevant to the protection of their health, with this lack of relevance creating a barrier to AQHI adoption. The child care provider in the higher SES area explained that currently with respect to AQHI: “*It doesn't feel like it's a priority”* since “*… you don't tend to get air quality emphasized as much in the media”.* Seniors in the lower SES area expanded on the need to communicate AQHI relevance by suggesting that AQHI engagement should: *“Get them to understand what it is that index is trying to accomplish and then to relate it to self …”*As well, parents of young children in the lower SES area stressed the need to communicate AQHI relevance since *“…people just don't have the importance of it.”*

#### Index confusion

Additionally, participants expressed index confusion between the AQHI and other indices as a barrier to AQHI adoption. Aside from the respiratory health care providers, confusion about what the AQHI was and how it differed from other indices such as the humidex (an index used in Canada that incorporates both heat and humidity to describe how hot the weather feels to the average person [35]) were expressed by the senior and child care providers as well as all at risk populations even after learning about the tool. For example the senior care provider in the higher SES area expressed: *“Because I always think of the pollution index. They used to always do the pollution index…But now they don't even talk about the pollution.”* Index confusion was also expressed by the child care provider in the higher SES area who commented: *“But that's — again that goes back to the heat.”* This same confusion was repeated by parents of young children in the lower SES area who asked: *“Oh that's the heat one?”* Seniors in the higher SES area summed up AQHI confusion by stating: *“Unfortunately [in] our society there are so many similar acronyms for different things depending on the field you’re in.”*

#### Sensory cue precedence

Moreover, participants expressed that they relied on sensory cues (i.e. feel, taste, sight) over real-time measured and reported air quality information to implement health protective behaviours, with this sensory cue precedence acting as a barrier to AQHI adoption. Aside from respiratory health care providers, all other participants emphasized that they mainly rely on sensory cues (i.e. feel, taste, sight) to implement health protective behaviours related to air quality. The senior care provider in the lower SES area indicated: *“It's like when I open the window and I don't feel good it's not a good time to go outside.”* This reliance on sensory cues was also expressed by the child care provider in the higher SES area who indicated: *“…I think it's very much personal cues…”* and the child care provider in the lower SES area who stated: *“…the staff go outside for a few minutes and they notice or they'll go on their lunch and they come back… you can't breathe outside…the air quality is not the greatest today, then we would definitely keep the children inside.”*

In addition, sensory cue precedence was expressed by people with existing respiratory conditions in the lower SES area who indicated: *“If it’s that hot out I’m not going out.”* Seniors in the lower SES area also voiced reliance on sensory cues by stating: *“You can taste what’s out there in that air.”* Also seniors in the higher SES expressed that: *“You can see the haziness in the air. You are able to see in the atmosphere.”* Similarly, parents of young children in the higher SES area stated: *“You just kind of go outside and you're like, yeah it feels okay out there.”*

#### Time constraints

An additional barrier to AQHI adoption expressed by participants includes time constraints. Aside from respiratory health care providers who visit the weather website to calibrate equipment to conduct their work, senior and child care providers indicated that their current work demands are not conducive to checking AQHI throughout the day. In addition, at risk populations also stressed the inconvenience of checking throughout the day. The senior care provider in the higher SES area indicated: *“So many things come down to just time.”* Likewise, the child care provider in the higher SES area indicated: *“…personally I don't have time in here for that”* and the child care provider in the lower SES area reiterated: *“…sometimes it's hard to do that because, you know, you're rushing to get to work.”* The inconvenience of checking AQHI information via the website was expressed by people with existing respiratory conditions in the higher SES area who indicated: *“I just don’t think many people want to go in and click 100 times to get to the thing…”*

### Strategies to increase AQHI uptake

#### Professional network promotion

A facilitator to AQHI adoption included AQHI promotion via professional networks. Health care and service providers indicated that they rely on their existing professional networks such as upper management and public health services for guidance regarding tools to protect the health of their at risk populations from exposure to air pollution. Supportive comments with respect to engaging upper management about AQHI such that they could pass on the information to staff were provided by the senior care provider in the lower SES area who indicated: *“I think meeting all the Directors of Nursing”* in reference to increasing AQHI implementation in practice. Additional supportive comments from the senior care provider in the higher SES area included: *“I always enjoy getting things from Public Health because they're usually good.”* As well, those with existing respiratory conditions in the higher SES area praised their respiratory care provider with guiding them and stated: *“I think someone like [respiratory health care provider] just telling you point blank this is your situation and this is what you have and you have to take care of it.”* Acknowledgement was also expressed by parents of young children in the lower SES area who indicated: *“And I mean being at the daycare they would always tell us the air quality.”*

#### Health benefit emphasis

The other strategy to increase AQHI uptake offered by participants included emphasis on the health benefits of AQHI adoption. The senior care provider in the higher SES area stressed the need to *“…explain the benefits from it [AQHI] too…”* such that the importance of using the tool would be clear. Seniors in the lower SES area expanded on the need to emphasize the benefits of the AQHI via clear communication by stating: *“If they said what AQHI meant.”* As well, the need to emphasize AQHI benefits was expressed by parents of young children in the lower SES area who suggested: “*If you tell me the importance of it and I grasp that, then I'm going to check no matter what*.”

#### Neighbourhood scale focus

Participants also expressed that AQHI information reported at a neighbourhood scale as a facilitator to AQHI adoption. Participants stressed the difference in air quality experienced above (higher SES, further from IC) and below (lower SES, closer to IC) the Niagara Escarpment, with the air quality ‘above’ the escarpment perceived as being more favourable than that below the escarpment. The child care provider in the lower SES area described these differences in air quality by stating: *“…when they come into or closer to the city, like the downtown area they find it's more congested.”* Likewise people with existing respiratory conditions in the lower SES area expressed: *“They are saying air quality but what about down the city and then the mountain… it’s so different.”* These differences in air quality were stressed again by parents in the lower SES area who stated: “*There's way more pollution here [downtown below escarpment].”* Additional support for AQHI information at a neighbourhood scale was expressed by the people with existing respiratory conditions in the higher SES area who reflected upon the current AQHI information and indicated: *That’s unsettling because they may say it’s 3 on theirs and my area might be higher…”*

#### Local media reporting

Participants expressed that local media reporting of AQHI as a strategy to increase AQHI uptake. Parents of young children in the lower SES area stated: *“…people do watch the news.”* Likewise, seniors in the lower SES area articulated that *“The radio in my opinion is better…”* and people with existing respiratory conditions in the lower SES area noted that *“It should be on the first page [newspaper].”*

#### Wearable device option

Participants suggested that providing AQHI information on wearable devices could act as a strategy to increase AQHI uptake. Wearable devices reporting current AQHI information were identified as being facilitators to AQHI adoption by people with existing respiratory conditions in the higher SES area. They noted that real-time AQHI information is critical for health protection and proposed: *“But what about some kind of a bracelet that we could wear and if the air quality gets bad our bracelet would change colour and we’d know get our[selves] in the house.”*

## Discussion

Since AQHI reporting in Hamilton first started during the summer of 2011, Ontario -wide reporting of AQHI has been implemented to communicate the health risks of outdoor air pollution. Therefore, adoption of the AQHI is critical to protection of population health from outdoor air pollution exposure, particularly for at risk populations and those caring for them. In this exploratory study, health care and service providers and their respective at risk populations not only expressed their level of AQHI knowledge but also provided insight into the factors influencing AQHI adoption and offered strategies that may increase AQHI uptake.

Our study found that AQHI knowledge centered on numerical reliance and health protective intent but varied with SES. This is consistent with our previous work on AQHI knowledge in Hamilton [36] which also highlighted that there was knowledge about the health protective intent of the AQHI but this knowledge varied with SES. Research points out that health literacy and numeracy (ability to use numerical health information to make appropriate decisions about health) are critical for health self-management which would include AQHI adoption [37]. Accordingly, understanding AQHI, which is expressed on a scale from 1 to 10 is critical to health protection and perceptions of health-related risk [38]. Moreover, as other studies have found [39] including our previous work assessing AQHI knowledge in Hamilton [36], this study found a higher level of AQHI knowledge among higher SES individuals. Although increasing AQHI knowledge is critical in all at risk populations, particular attention must be given to seniors living in lower SES areas suffering from co-morbidities [40]. In the US, higher rates of limited health literacy (ability to use health information to make appropriate decisions about health) were found in those of lower SES and the elderly [41].

Increasing AQHI knowledge among the at risk populations could be achieved via AQHI promotion by their respective health care and service providers. Professional networking via social media sites for health care professionals provides an opportunity to communicate about patient issues in a protected forum [42]. Therefore, increases in AQHI knowledge could be fostered by AQHI promotion among health care and service providers via social media sites [43]. In turn, health care and service providers would be able to transfer AQHI knowledge to their respective at risk populations [12].

Not only is knowledge instrumental with respect to AQHI adoption, but so are the characteristics of the AQHI. In line with Rogers [14], because the relative advantage of the AQHI was not clear to service providers and the public, the benefits in terms of decreasing adverse health effects due to air pollution exposure were difficult to perceive and AQHI was not adopted by the majority of participants. Therefore, improving effectiveness of AQHI messages such that they reach at risk populations and those caring for them to persuade behaviour change can be achieved by emphasizing the health benefits of the AQHI [44].

Due to geographical variability and the inability of the AQHI to capture air quality and health information in real-time at a neighbourhood scale, uncertainty in AQHI information was experienced. Consistent with our previous work [36], sensory cues (i.e. feel, see, taste) were preferred over AQHI information to guide health protective behaviour. Therefore, AQHI information reported at a neighbourhood scale would assist in addressing this uncertainty which may in turn decrease the likelihood of sensory cues being used solely to guide health protective behaviour in response to air pollution exposure [45]. Consequently, health care and service providers would be less inclined to implement health protective behaviours for their respective at risk populations based on their own sensory cues which may differ from that of their at risk populations. Health care and service providers’ adoption of AQHI without reliance on sensory cues is critical to the protection of at risk populations and promoting health protective behaviour.

The most common reported barrier influencing AQHI adoption included time constraints. Consistent with what health care providers such as physicians [46] and nurses [47] have reported with respect to implementing new innovations in practice, time constraints were the most commonly reported barriers to AQHI adoption by health care and service providers in our study. Likewise, time constraints were the most common barrier reported by the population with respect to engaging in health protective behaviours including physical activity [48] and vaccination [49]. By reporting AQHI information on local media (i.e. television, radio, newspaper) and providing a wearable device option [50] at risk populations and those caring for them would have access to AQHI information all the time with little effort.

### Limitations

Response bias would imply that health care and service providers and at risk populations who participated were likely to be interested in AQHI. Another limitation is that our methodology involved a time gap of over 3 years between the focus group discussions. We experienced challenges in recruitment of lower SES at risk populations with existing respiratory conditions (i.e. asthma). Consequently, our methodology involved a comparison of groups with asthma and chronic obstructive pulmonary disease (COPD) as existing respiratory conditions. This delay could have impacted the factors explored in this study; however, no new information was attained from the COPD focus group. Additionally, due to a malfunctioning recorder, one interview was not recorded and transcribed; only notes were taken.

Our study only included one health care and service provider from the lower and higher SES areas, respectively. Given our preference to recruit health care providers that were working directly with at risk populations, we did not recruit specialists such as cardiologists or respiratory physicians working in the City. Consequently, we did have a small sample of health care and service providers in our study. However, all participants including the at risk populations were asked the same questions via two different data collection methods, ensuring data triangulation. Because triangulation can be used to explore one phenomenon from different points and perspectives, it propels towards data saturation [51]. By using this approach, no new information was attained since similar responses were provided again and again [52].

### Implications for research

The Diffusion of Innovations model was useful in explaining health care and service providers’ and their respective at risk populations’ decision to adopt the AQHI. We incorporated the determinants of health framework by examining health care and service providers’ (organization) and their at risk populations’ (community) adoption of the AQHI in lower and higher SES areas. Further research should bridge AQHI adoption at the individual, organization and community level with a “determinants of health” lens in order to develop a comprehensive approach.

### Implications for practice

Intervention strategies to increase AQHI knowledge and encourage adoption by at risk populations in lower SES areas should be considered as upstream public health measures designed to offset potentially significant downstream costs.

## Conclusions

Our exploratory qualitative study highlighted that AQHI knowledge, AQHI characteristics and perception of air quality and health were critical to AQHI adoption. By increasing AQHI knowledge, emphasizing AQHI relevance, and reporting AQHI information at a neighbourhood scale via local media sources and wearable devices, increases in AQHI uptake can be achieved while accounting for SES differences.

## Ethics approval and consent to participate

This research received ethics approval from McMaster University Research Ethics Board. Certificate of Ethics Clearance to Involve Human Participants in Research Project Number: 2012 109.

## Availability of data and materials

Signed confidentiality agreements prevent us from sharing the data.
